# Inhibition of TNF in the Brain Reverses Alterations in RAS Components and Attenuates Angiotensin II-Induced Hypertension

**DOI:** 10.1371/journal.pone.0063847

**Published:** 2013-05-15

**Authors:** Srinivas Sriramula, Jeffrey P. Cardinale, Joseph Francis

**Affiliations:** 1 Comparative Biomedical Sciences, School of Veterinary Medicine, Louisiana State University, Baton Rouge, Louisiana, United States of America; 2 Department of Pharmacology and Experimental Therapeutics and Cardiovascular Center of Excellence, Louisiana State University Health Sciences Center, New Orleans, Louisiana, United States of America; University of Illinois at Chicago, United States of America

## Abstract

Dysfunction of brain renin-angiotensin system (RAS) components is implicated in the development of hypertension. We previously showed that angiotensin (Ang) II-induced hypertension is mediated by increased production of proinflammatory cytokines (PIC), including tumor necrosis factor (TNF), in brain cardiovascular regulatory centers such as the paraventricular nucleus (PVN). Presently, we tested the hypothesis that central TNF blockade prevents dysregulation of brain RAS components and attenuates Ang II-induced hypertension. Male Sprague-Dawley rats were implanted with radio-telemetry transmitters to measure mean arterial pressure (MAP) and subjected to intracerebroventricular (ICV) infusion of etanercept (10 µg/kg/day) with/without concurrent subcutaneous 4-week Ang II (200 ng/kg/min) infusion. Chronic Ang II infusion resulted in a significant increase in MAP and cardiac hypertrophy, which was attenuated by inhibition of brain TNF with etanercept. Etanercept treatment also attenuated Ang II-induced increases in PIC and decreases in IL-10 expression in the PVN. Additionally, Ang II infusion increased expression of pro-hypertensive RAS components (ACE and AT_1_R), while decreasing anti-hypertensive RAS components (ACE2, Mas, and AT_2_ receptors), within the PVN. ICV etanercept treatment reversed these changes. Ang II-infusion was associated with increased oxidative stress as indicated by increased NAD(P)H oxidase activity and super oxide production in the PVN, which was prevented by inhibition of TNF. Moreover, brain targeted TNF blockade significantly reduced Ang II-induced NOX-2 and NOX-4 mRNA and protein expression in the PVN. These findings suggest that chronic TNF blockade in the brain protects rats against Ang II-dependent hypertension and cardiac hypertrophy by restoring the balance between pro- and anti-hypertensive RAS axes and inhibiting PIC and oxidative stress genes and proteins in the PVN.

## Introduction

The renin-angiotensin system (RAS) plays a central role in the development and regulation of blood pressure response. Angiotensin II (Ang II), the effector peptide of the pro-hypertensive axis of the RAS that also includes angiotensin converting enzyme (ACE) and the Angiotensin -type 1 receptor (AT_1_R), exerts diverse physiological actions in both the peripheral and central neural systems [1], [2]. The anti-hypertensive counterbalance to these mediators includes ACE2, Ang-(1–7) and the Mas receptor [2]. Importantly, all these essential components of the RAS, including renin and angiotensinogen, as well as various cardiovascular-modulatory aminopeptidases, are synthesized within the brain, suggesting the existence of a comprehensive intrinsic brain RAS [3]–[6]. Recent evidence suggests that dysregulation of the individual brain RAS axes may play a critical role in the development and maintenance of hypertension [2], [7]. Ang II, acting through the AT_1_R, plays a prominent role in the central regulation of blood pressure by activating the sympathetic nervous system, regulating fluid and salt balance and the secretion of aldosterone, amongst other actions [8]. Previous studies suggest that systemically delivered Ang II likely acts upon the circumventricular organs, where the blood brain barrier is weak or absent, and subsequently activates hypothalamic and brain stem sites such as the paraventricular nucleus (PVN) and ventrolateral medulla, contributing to sympathoexcitation and hypertensive response [9], [10]. Experimental evidence indicates that the hypothalamic PVN is an important center for integrating Ang II-induced neural outflow signals for the pressor response and sympathetic vasomotor tone [7], [11], [12].

Recent findings from our lab and others suggest that the RAS, in addition to inducing neurohumoral excitation, also increases the production of proinflammatory cytokines (PICs), such as tumor necrosis factor-alpha (TNF), in brain cardiovascular regulatory centers, and has been shown to contribute to the neurogenic component of hypertension, both through direct actions and through modulating reactive oxygen species (ROS) signaling pathways [13]–[16]. A chronic increase in peripheral Ang II levels is proposed to initiate a cascade of signaling events involving PICs and ROS in brain cardioregulatory sites raising sympathetic activity, hypertension and end organ damage. A study by Marvar et al. showed that Ang II-mediated hypertension is caused by central mechanisms and described a feed-forward process in which the central pressor effects of Ang II lead to activation of T cells, which in turn, promote vascular inflammation and further raise blood pressure, leading to severe hypertension [17]. In addition, PICs can be produced locally in the brain by glia and neurons, thereby contributing to the neuroinflammatory response implicated in the pathogenesis of hypertension [14]. These observations, coupled with the emerging role of PICs and the little known role of the anti-hypertensive axis of the RAS in hypertension, led to hypothesize that the central effects of Ang II are, at least in part, mediated by the activation of PICs, especially TNF. The resultant of these actions are the differentially dysregulated RAS axes within cardiovascular relevant brain regions, including the PVN, ultimately enhancing the neurogenic hypertensive response. In the current study, we investigate this hypothesis by examining central TNF inhibition via intracerebroventricular (ICV) etanercept infusion, a soluble TNF receptor fusion protein, on pro- and anti-hypertensive RAS components in the PVN in Ang II-induced hypertension.

## Materials and Methods

### Ethics Statement

All animal and experimental procedures in this study were reviewed and approved by the Institutional Animal Care and Use Committee (IACUC) at Louisiana State University in compliance with National Institutes of Health Guide for the Care and Use of Laboratory Animals.

### Experimental Design

Male Sprague-Dawley rats (10–12 weeks old) were used in this study. Animals were housed in a temperature-controlled room (25±1°C) and maintained on a 12∶12 hour light:dark cycle with free access to food and water. The rats were implanted with radio-telemetry transmitters to measure blood pressure, and subjected to ICV infusion of etanercept (ETN; 10 µg/kg/day) or artificial cerebrospinal fluid (aCSF) (Alzet, model 1004; 0.11 µl/hr), with and without subcutaneous infusion of Ang II (200 ng/kg/min) for 4 weeks. Osmotic minipumps (Alzet, model 2004; 0.25 µl/hr) were filled with Ang II dissolved in 0.9% saline or saline alone, and were implanted subcutaneously in the retroscapular area. The rats were divided into 4 groups: 1) Control group: saline minipumps+ICV aCSF, 2) ETN group: saline minipumps+ICV etanercept, 3) Ang II group: Ang II minipump+ICV aCSF, and 4) Ang II+ETN group: Ang II minipump+ICV etanercept. At the end of the study, rats were euthanized; the hearts and brains were collected and stored at −80°C until further analysis.

### Blood Pressure Measurement

Blood pressure was measured continuously in conscious rats implanted with radio-telemetry transmitters (Model TA11PA-C40, Data Sciences International, St. Paul, MN). Rats were anesthetized with a ketamine (90 mg/kg) and xylazine (10 mg/kg) mixture (i.p.) and placed dorsally on a heated surgical table. The adequacy of anesthesia was monitored by limb withdrawal response to toe pinching. An incision was made on the ventral surface of the left leg, and the femoral artery and vein were exposed and dissected apart. The femoral artery was ligated distally, and a small clamp was used to temporarily interrupt the blood flow. The catheter tip was introduced through a small incision in the femoral artery, advanced into the abdominal aorta such that the catheter tip was distal to the origin of the renal arteries, and secured into place. The body of the transmitter was placed into the abdominal cavity and secured to the abdominal wall. The abdominal musculature was sutured and the skin layer was closed following implantation. Rats received benzathine penicillin (30000 U, i.m.) and buprenorphine (0.1 mg/kg, s.c.) immediately following surgery and 12 h postoperatively and allowed to recover for seven days.

### ICV Cannula Implantation

Following the transmitter recovery period, the rats were implanted with ICV cannula for infusion of etanercept or aCSF [18]. The rats were anesthetized with ketamine (90 mg/kg) and xylazine (10 mg/kg) mixture (i.p.) and the head was positioned in a Kopf stereotaxic apparatus. An ICV cannula was implanted into the right lateral cerebroventricle (1.3 mm caudal to bregma, 1.5 mm lateral to the midline, and 3.5 mm ventral to the dura) according to Paxinos and Watson, and fixed to the cranium using small screws and dental cement. A 4-week osmotic minipump was implanted subcutaneously and connected to the infusion cannula via the catheter tube to deliver etanercept or aCSF into the brain.

### Measurement of Plasma IL-10

At the end of the study, blood was collected in chilled EDTA tubes; plasma was separated and stored at −80°C until assayed. Circulating levels of IL-10 were quantified in the plasma using a commercially available rat IL-10 ELISA kit (Invitrogen) according to manufacturer’s instructions.

### Real Time RT-PCR

The PVN punches were made from frozen brain sections using a Stoelting brain punch (Stoelting). Total RNA was isolated from PVN tissue using RNeasy plus micro kit (Qiagen) and cDNA was synthesized using iScript cDNA synthesis kit (Bio-Rad). Real Time PCR amplification reactions were performed with iQ SYBR Green Super mix with ROX (Bio-Rad) using the ABI Prism 7900 Real time PCR machine (Applied Biosystems). Data were normalized to GAPDH expression by the ΔΔC_T_ comparative method.

### Western Blot Analysis

Western blot analysis was performed according to standard protocols. The PVN tissue was homogenized with RIPA lysis buffer. The protein concentration was measured using a bicinchioninic acid protein assay kit (Pierce). Equal amounts of protein (5 µg) were separated by SDS-PAGE on 10% or 12% gels, transferred on to PVDF membrane (Immobilon-P, Millipore), and blocked with 1% BSA in TBS-T at room temperature for 60 min. The membranes were subjected to immunoblot analyses with anti-ACE (Santa Cruz, 1∶500), anti-AT_1_R (Santa Cruz, 1∶500), anti-ACE2 (Santa Cruz, 1∶500), anti-AT_2_R (Santa Cruz, 1∶200), anti-Mas (Alomone Labs, 1∶500), anti-NOX-2 (BD Biosciences, 1∶500), anti-NOX-4 (Santa Cruz, 1∶1000), anti-iNOS (Santa Cruz, 1∶500), anti-nNOS (Santa Cruz, 1∶500), and anti-GAPDH (Santa Cruz, 1∶1000) antibodies. The membranes were washed and incubated with anti-rabbit or anti-goat secondary antibodies (Santa Cruz, 1∶5000) for 1 hour at room temperature. Specific bands were detected using an enhanced chemiluminescence kit (Amersham). The bands were quantified by densitometry using Chemidoc XRS system and Quantity-One software (Bio-Rad) and were normalized to GAPDH expression.

### Determination of NAD(P)H Oxidase Activity in the PVN

Homogenates were prepared from PVN samples and total protein concentration was determined using a bicinchoninic acid protein assay kit (Pierce). The NAD(P)H oxidase activity was measured using lucigenin enhanced chemiluminiscence detection of superoxide as previously described [19]. In brief, the homogenates were diluted in modified HEPES buffer containing 140 mM NaCl, 5 mM KCl, 0.8 mM MgCl_2_, 1.8 mM CaCl_2_, 1 mM Na_2_ HPO_4_, 25 mM HEPES, and 1% glucose (pH 7.0). The reaction was started by addition of NAD(P)H (100 µM) and dark adapted lucigenin (5 µM). Light emission was recorded and expressed as mean light unit (MLU) per minute per milligram of protein over 10 min. Using this method, the superoxide anion production also represents NAD(P)H oxidase activity. The specificity of superoxide measured was confirmed either by adding superoxide dismutase (200 units/ml) or apocynin (1 mM).

### *In situ* Detection of Superoxide Production in the PVN

Dihydroethidium, an oxidative fluorescent dye, was used to detect in situ superoxide in the PVN of rats as previously described [19]. At the end of the experiment, the brains were removed, quickly frozen, embedded into OCT, and cryostat sectioned (30 µm, coronal) directly onto chilled microscope slides. Sections were then incubated in a light protected humidified chamber at 37°C for 30 minutes with 1 µmol/L dihydroethidium (Molecular Probes). After washing with phosphate-buffered saline, red fluorescence was visualized by confocal laser scanning microscopy using an excitation wavelength of 490 nm and emission wavelength of 610 nm.

### Statistical Analysis

All results are expressed as mean±SEM. For statistical analysis of the data, Student’s *t* test, one-way ANOVA or repeated measures ANOVA followed by Bonferroni’s *post hoc* test was performed using GraphPad Prism version 5.0 for Windows (Graph Pad Software, San Diego California, USA) to determine differences among groups. A value of *p*<0.05 was considered statistically significant.

## Results

### Effect of Central TNF Blockade on Mean Arterial Pressure and Cardiac Hypertrophy

**Figure 1:**
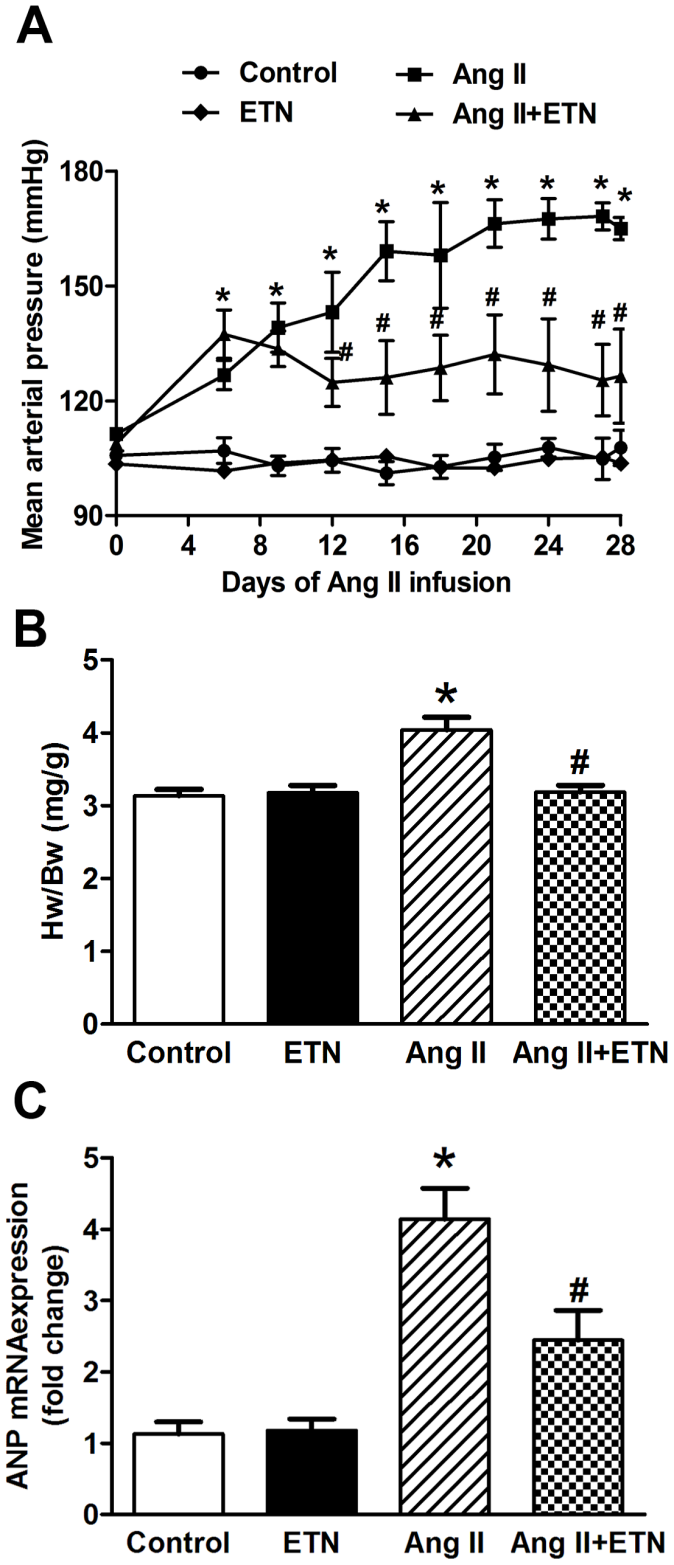
Effect of central TNF blockade on mean arterial pressure (MAP) and cardiac hypertrophy. Ang II infusion for 28 days significantly increased the MAP versus control group, which was attenuated by central blockade of TNF using etanercept (**A**). Chronic Ang II infusion resulted in increased cardiac hypertrophy, as assessed by the ratio of heart weight to body weight (HW/BW) (**B**) and mRNA expression of atrial natriuretic peptide (ANP) in the left ventricular tissue (**C**), which was reduced following central etanercept treatment. n = 10–12 per group, *p<0.05 vs Control, #*p*<0.05 vs Ang II.

To assess the effect of central TNF blockade on the Ang II-induced hypertensive response, mean arterial pressure (MAP) was measured using a radio-telemetry system. After 28 days, chronic Ang II infusion significantly increased the MAP in rats when compared with control rats (165±5 mmHg vs 108±8 mmHg, respectively; *p*<0.05) (Figure 1A). In contrast, ICV treatment with etanercept attenuated the Ang II-induced increase in MAP (126±21 mmHg vs 165±5 mmHg, respectively; *p*<0.05), while etanercept treatment alone had no effect on MAP (104±4 mmHg).

To evaluate Ang II-induced changes on cardiac hypertrophy in these rats, the hearts were harvested and weighed at the end of experimental period. The ratio of heart weight/body weight (HW/BW) was calculated as an indicator of cardiac hypertrophy. Chronic Ang II infusion lead to increased cardiac hypertrophy versus controls as indicated by the increased HW/BW ratio (Figure 1B). ICV treatment with etanercept inhibited Ang II-mediated cardiac hypertrophy. Furthermore, mRNA expression of a molecular marker of cardiac hypertrophy, atrial natriuretic peptide (ANP), was measured in cardiac tissue using real time RT-PCR. Chronic Ang II infusion showed increased mRNA expression of ANP in the heart, which was decreased by ICV treatment with etanercept (Figure 1C). These data suggests a role for TNF in the brain on Ang II-induced blood pressure regulation and cardiac hypertrophy in the hypertensive state.

### Effect of Central TNF Blockade on the Expression of Pro- and Anti-inflammatory Cytokines

**Figure 2:**
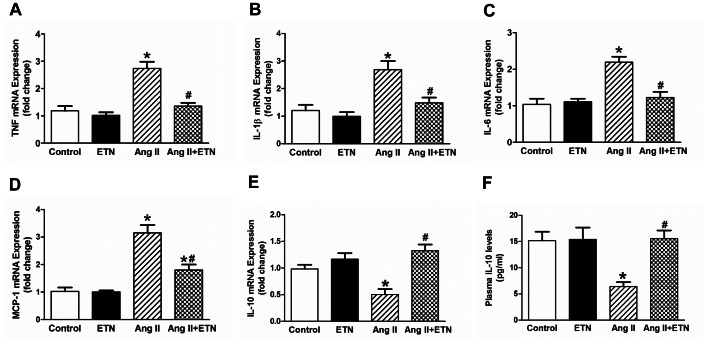
Effect of central TNF blockade on pro-and anti-inflammatory cytokine expression. Chronic Ang II infusion significantly increased the expression of TNF (**A**), IL-6 (**B**), IL-1β (**C**), and the chemokine MCP-1 (**D**) in the PVN. This increase in PIC expression was attenuated in rats treated with etanercept. Furthermore, Ang II infusion significantly decreased the mRNA expression of the anti-inflammatory cytokine IL-10 in the PVN (**E**) and plasma (**F**) compared with controls. This decrease was restored by central TNF blockade with etanercept. n = 8–10 per group, *p<0.05 vs Control, #*p*<0.05 vs Ang II.

To determine the effect of Ang II on the production of PICs and chemokines, the mRNA expression of TNF, IL-6, IL-1β, and the chemokine MCP-1 were measured in the PVN by real time RT-PCR (Figures 2A, B, C, and D). Ang II infusion induced an increase in the gene expression of the PICs TNF, IL-6 and IL-1β, and the chemokine MCP-1, in the PVN, when compared to the control group. These PICs were attenuated in the PVN of Ang II-infused rats treated ICV with etanercept, demonstrating that chronic Ang II infusion increases the pro-inflammatory response within the PVN through TNF in Ang II-induced hypertension.

We also examined the effect of TNF inhibition on the expression of the anti-inflammatory cytokine IL-10. Chronic subcutaneous Ang II infusion resulted in significant decrease in PVN mRNA and plasma protein levels of IL-10. However, Ang II infusion and simultaneous inhibition of TNF in the brain using etanercept restored the PVN mRNA expression of IL-10 (Figure 2E) and plasma levels of IL-10 (Figure 2F).

### Effect of Central TNF Blockade on the Expression of RAS Components in the PVN

**Figure 3:**
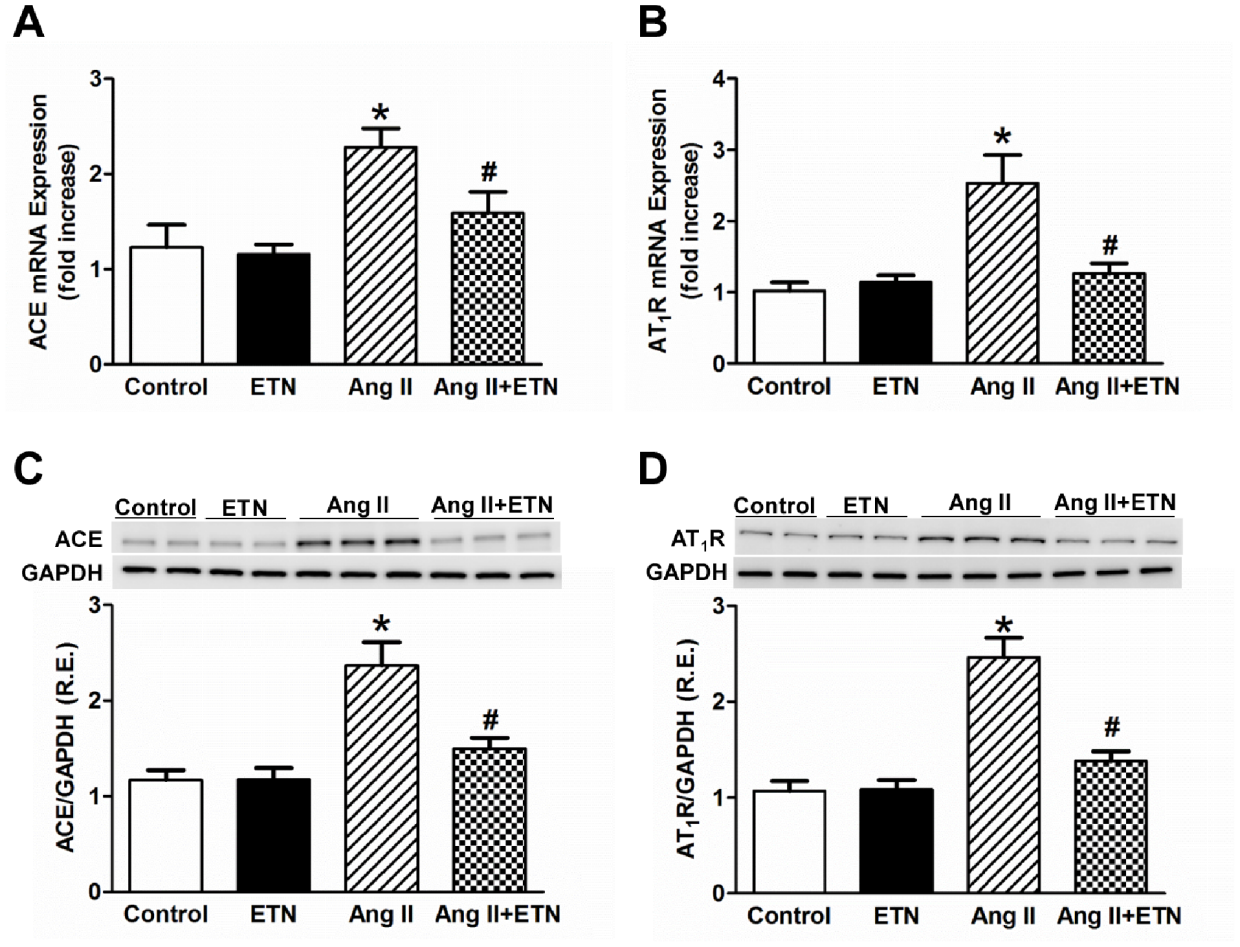
Effect of central TNF blockade on pro-hypertensive RAS component expression in the PVN. Chronic Ang II infusion for 4 weeks significantly increased the mRNA and protein expression of pro-hypertensive components, ACE and AT_1_R in the PVN, which was attenuated in Ang II+ETN rats. R.E. Relative expression, n = 8–12 per group, *p<0.05 vs Control, #*p*<0.05 vs Ang II.

**Figure 4:**
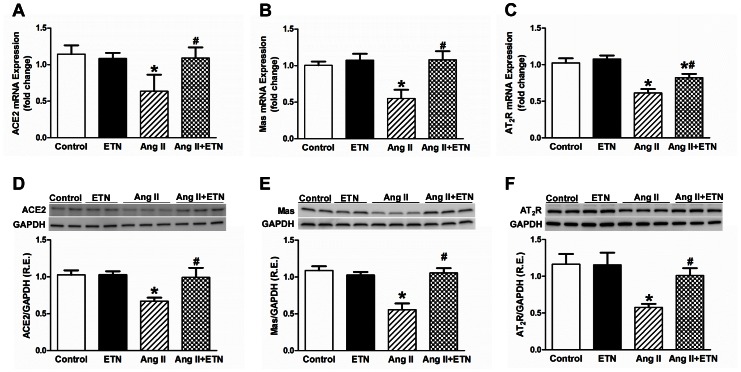
Effect of central TNF blockade on anti-hypertensive RAS component expression in the PVN. Chronic 28-day Ang II infusion decreased the mRNA and protein expression of anti-hypertensive RAS components, ACE2, the Mas receptor, and AT_2_ receptors in the PVN. This decrease was prevented by central TNF blockade using etanercept. R.E. Relative expression, n = 8–12 per group, *p<0.05 vs Control, #*p*<0.05 vs Ang II.

Both Ang II and TNF have been shown to modulate RAS component expression. To determine the manner by which Ang II infusion alters the expression of the pro- and anti-hypertensive components of the RAS in the PVN, we examined the mRNA and protein expression levels of ACE, ACE2, AT_1_R, AT_2_R, and the Mas receptor. The PVN mRNA and protein expression of the RAS pro-hypertensive components ACE and AT_1_R were significantly increased in Ang II-infused rats when compared with control rats; this was prevented by ICV treatment with etanercept (Figure 3). Conversely, the anti-hypertensive components of the RAS (ACE2, Mas receptor and AT_2_R) showed a decreased gene and protein expression in Ang II-treated rats. These levels were increased in the Ang II+ETN group (Figure 4). These data suggest that in Ang II-induced hypertension, the pro- and anti-hypertensive components of the RAS are differentially regulated within the PVN in a deleterious manner and TNF inhibition restores the balance between these RAS components.

### Effect of Central TNF Blockade on the Oxidative Stress Markers in the PVN

**Figure 5:**
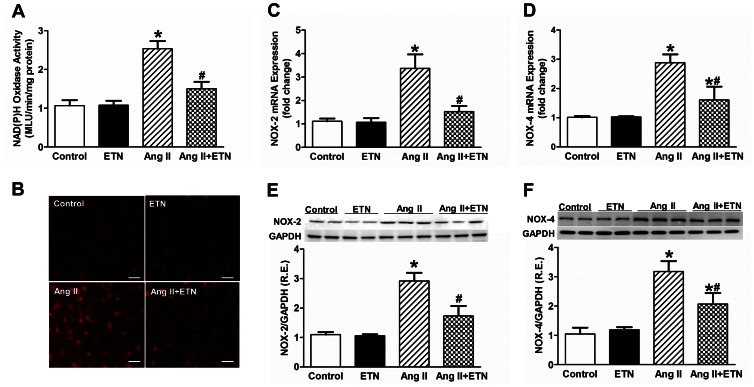
Effect of central TNF blockade on oxidative stress markers in the PVN. Ang II-infused rats demonstrated an increase in the NAD(P)H oxidase activity (**A**) and superoxide production (**B**) in the PVN compared with vehicle treated rats. These increases were reduced by TNF blockade with etanercept following Ang II infusion. Also, brain targeted TNF blockade significantly reduced Ang II-induced NOX-2 and NOX-4 mRNA (**C** and **D**) and protein (**E** and **F**) expression in the PVN. R.E. Relative expression, n = 8–12 per group, *p<0.05 vs Control, #*p*<0.05 vs Ang II.

**Figure 6:**
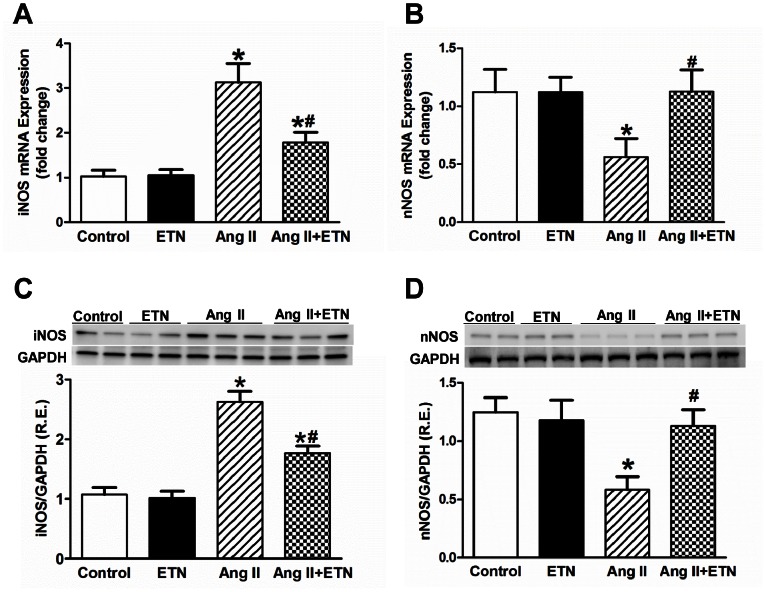
Effect of central TNF blockade on iNOS and nNOS mRNA and protein expression in the PVN. Ang II-infused rats demonstrated an increase in the mRNA and protein expression of iNOS, and decrease in expression of nNOS within the PVN. These alterations were reversed by TNF blockade with ICV etanercept treatment. R.E. Relative expression, n = 8–12 per group, *p<0.05 vs Control, #*p*<0.05 vs Ang II.

Ang II and TNF have both been shown to act through oxidative stress mediated pathways, especially within the PVN, in inducing elevated sympathetic outflow and a progressive hypertensive response. Thus, several experiments were performed to test the effect of central TNF inhibition on oxidative stress markers in the PVN in response to the Ang II infusion hypertension model. First, NAD(P)H oxidase dependent superoxide production in the PVN was measured using a lucigenin enhanced chemiluminiscence method. Consistent with earlier reports, Ang II caused a significant increase in NAD(P)H oxidase dependent superoxide production in the PVN homogenates compared with control rats. This response was abolished by ICV inhibition of TNF using etanercept (Figure 5A). Next, in situ dihydroethidium fluorescence was used to assess Ang II-induced superoxide production in the PVN of rats receiving systemic Ang II-infusions concomitant with ICV infusions of either etanercept or aCSF. Consistent with earlier reports, dihydroethidium fluorescence was increased in the PVN of Ang II-infused rats compared with control rats. This response was markedly inhibited in rats receiving ICV etanercept (Figure 5B). Furthermore, Ang II infusion significantly increased the NOX-2 and NOX-4 mRNA (Figures 5C and D) and protein expression (Figures 5E and F) in the PVN of Ang II infused rats. These changes were attenuated in Ang II-infused rats treated centrally with etanercept. To further determine the effect of TNF blockade on Ang II-induced NO signaling, we analyzed mRNA and protein expression of inducible NOS (iNOS) and neuronal NOS (nNOS) within the PVN. Four weeks of Ang II infusion significantly increased iNOS mRNA and protein expression and decreased the nNOS mRNA and protein expression. These changes were prevented in rats with central infusion of etanercept (Figure 6). Overall, these data suggest that increased oxidative stress in Ang II-induced hypertension is potentially through a TNF-driven mechanism and the effects of central TNF blockade on attenuation of Ang II-induced hypertension, at least in part, are mediated by a decrease in oxidative stress.

## Discussion

In the present study, the role of central TNF on Ang II-induced hypertension and cardiac hypertrophy was investigated. These data suggest for the first time that inhibition of TNF in the brain using etanercept lowered blood pressure, reduced inflammation, decreased oxidative stress, and restored the balance between the pro- and anti-hypertensive axes of the RAS. Our findings demonstrate that the changes observed in Ang II-induced hypertension are regulated, at least in part, through the central actions of TNF and potentially via the dysregulation of components of the RAS within the hypothalamic PVN.

Recent evidence suggests that hypertension is an inflammatory condition where various PICs such as TNF, IL-6 and IL-1β, both centrally and peripherally, have been shown to play an important role in the pathogenesis of hypertension [13], [14], [20], [21]. A recent study from our lab demonstrated that chronic peripheral Ang II infusion results in increased production of PICs within the PVN [15]. Etanercept is a soluble recombinant fusion protein that inhibits TNF by posing as a TNF receptor decoy and acting through competitive inhibition of TNF and an overall reduction in free TNF to act on endogenous receptors [22]. Blockade of TNF by etanercept has been shown to prevent renal damage in a genetic hypertensive rat model, as well as lower blood pressure in rats with Ang II- and salt-induced hypertension, suggesting a role for TNF in blood pressure regulation and renal injury [23], [24]. Another study also showed that mice treated with etanercept had an attenuated hypertension and a blunted increase in superoxide production in response to Ang II [25]. In our study, using ICV etanercept infusion techniques, we inhibited the TNF levels specifically in the brain. Our present observations complement those prior findings and show that blockade of TNF by ICV administration of etanercept into the brain protects rats against Ang II-dependent cardiac hypertrophy and hypertension.

Ang II can act as a potent proinflammatory agent and stimulate the production of chemokines such as MCP-1, and PICs, such as TNF, IL-6 and IL-1β in the brain [14], [21]. TNF is commonly considered as one of the initiators of the pro-inflammatory cascade, which can induce production of other cytokines, and inhibition of its action during inflammatory events abrogates many of the ensuing responses, including the production of IL-1β and IL-6 [26], [27]. A recent study by Shi et al. demonstrated that Ang II-induced hypertension involves activation of microglia and increased expression of PICs within the PVN [14]. A previous study from our group demonstrated that chronic Ang II infusion increases proinflammatory cytokines expression in the PVN of rats [21]. In our study, TNF blockade with etanercept decreased the PVN expression not only of TNF, but of other PICs such as IL-6, IL-1β, and the chemokine MCP-1, supporting the hypothesis that PICs are involved in the Ang II-induced hypertensive response. It has been shown that central gene transfer of IL-10 reduces hypothalamic inflammation in heart failure rats after myocardial infarction [28]. In addition, IL-10 overexpression in the PVN attenuates Ang II-induced hypertension [14]. In our study, chronic Ang II infusion resulted in decreased mRNA and protein expression of anti-inflammatory cytokine IL-10, which was restored by central TNF blockade suggesting that some of beneficial effects of TNF blockade are mediated by restoring the levels of anti-inflammatory IL-10.

Neurogenic hypertension is characterized by an overactive brain RAS [1], [2], [7]. In addition to locally generated Ang II within the brain, blood borne Ang II can enter the brain via circumventricular organs and modulate the pathogenesis of hypertension and fluid homeostasis [9], [10]. Elevated activity and expression of RAS components in central cardiovascular regulatory regions has been shown to be involved in the pathogenesis of hypertension in several genetic and experimental models [1]. Treatment with ACE inhibitors and Ang receptor blockers has been shown to prevent this RAS overactivity and restore normal cardiovascular function [1]. In the brain, the AT_1_R mediates the central effects of Ang II, including vasopressin release, water and salt intake and balance, and increased sympathetic drive, all of which contribute to the development of hypertension [29]. In many animal models of hypertension, the expression of the AT_1_R is up-regulated in central cardiovascular regulatory centers, including the hypothalamic PVN [7], [23]. Both *in vitro* and *in vivo* studies have demonstrated a cross-talk between Ang II and TNF [30], [31], a mechanism we have also shown using TNF knockout mice where Ang II-induced hypertension was attenuated via a decreased expression of AT_1_R [20]. Within the last decade, the discovery of an alternate set of components of the RAS which may act as a counterbalance to the actions of the ACE/Ang II/AT_1_R pathway added complexity to the understanding of RAS regulation, especially within the brain. These components, termed anti-hypertensive due to their cardio-protective effects, along with all the components of the pro-hypertensive RAS axis, are known to be expressed throughout the various central cardio-regulatory regions, including the PVN. In various experimental hypertensive models, the components of anti-hypertensive RAS axis (ACE2, Ang (1–7), the Mas receptor and AT_2_ receptors) are shown to be down-regulated, while the pro-hypertensive components are increased [15], [26], [29], [32]. This dysregulation may be the lynch pin trigger towards developing the hypertensive state, but it remains poorly understood. It has been shown that the brain ACE2 activity was inhibited in a chronically hypertensive mouse model with high Ang II levels and this decrease was mediated by AT_1_R [33]. We previously showed that ACE2 overexpression within the PVN attenuated Ang II-induced hypertension by abolishing PIC production in the PVN in combination with restoring the balance between pro- and anti-hypertensive axes of the RAS [15]. In the present study, ICV treatment with etanercept resulted in reduction of Ang II-induced pro-hypertensive RAS components expression in the PVN, including ACE and AT_1_R, as well as the restoration of the anti-hypertensive RAS components ACE2, Mas receptor, and AT_2_ receptors. These results suggest that this RAS dysregulation and perpetuation of the hypertensive state may be the result of a pro-inflammatory response through the actions of TNF.

Excessive ROS production in brain cardio-regulatory centers such as the PVN can contribute to the neurogenic component of the hypertensive response by enhancing sympathetic activity and outflow [34]. It has been previously shown that NAD(P)H oxidase is the primary source of Ang II-induced ROS in neurons and that treatment with Tempol, a cell permeable superoxide dismutase (SOD) mimetic, inhibits Ang II-mediated superoxide production and hypertension [35]. Enhanced NAD(P)H oxidase activity associated with increased expression of several NAD(P)H oxidase subunits including NOX-2 and NOX-4, have been shown to be predominant homologues expressed in the forebrain, including the PVN [36]. A recent study showed that in an aldosterone/salt induced hypertensive animal model, both NOX-2 and NOX-4 are necessary to generate functional NAD(P)H oxidase within the PVN [37]. Furthermore, TNF can induce activation of NADPH oxidase leading to enhanced oxidative stress and decreased bioavailability of NO [26]. In the present study, we found that central TNF inhibition abolished the Ang II-induced oxidative stress as indicated by decreased super oxide production, NAD(P)H oxidase activity as well as attenuated NOX-2 and NOX-4 subunit expression within the PVN (Figure 5). These results indicate that TNF mediates NAD(P)H oxidase-derived superoxide production during Ang II-induced hypertension. In addition, the decreased nNOS expression and increased iNOS expression in the PVN indicates NO signaling dysregulation in Ang II-induced hypertensive rats. Neuronal NOS is an inverse indirect indicator of sympathoexcitation, in that a decrease in nNOS correlates with an increase in sympathetic outflow. Therefore, a reduction in beneficial NO not only decreases with the reduction in nNOS expression and activity, but also due to the rapid interconversion of NO to peroxynitrite (ONOO^−^) due to the increased production of NADPH oxidase-derived superoxide. This is further complicated by the involvement of iNOS, which rapidly uses up available L-arginine for NO production and commences with superoxide production and thereby increasing free radical concentrations. This combination of factors can lead to an increased PIC response, sympathoexcitation and a continued propagation of neurogenic hypertension [16], [38]. The present study provides further support for these observations by showing that central TNF blockade with etanercept results in reduced oxidative stress within the PVN of hypertensive rats.

In summary, chronic Ang II infusion resulted in cardiac hypertrophy and elevated MAP, and within the PVN, an increased expression of PICs and markers of oxidative stress. More importantly, Ang II-infused rats had an increased expression of the injurious pro-hypertensive RAS components ACE and AT_1_R, and a decreased expression of the protective anti-hypertensive RAS components ACE2, the Mas receptor and AT_2_ receptors. These findings suggest that elevated TNF levels by Ang II hypertension are associated with initiation of inflammatory cascade, which in turn, promote downstream events and further raise blood pressure, leading to severe hypertension. Central blockade of TNF with etanercept resulted in attenuation of hypertension, cardiac hypertrophy and PIC expression, decreased oxidative stress, as well as a restored the balance between the protective and deleterious axes of the RAS, within the hypothalamic PVN. The beneficial effects of central TNF blockade in Ang II-induced hypertensive responses appears to be mediated by the returned balance of the central RAS components, especially within the PVN. It is important to note, however, that due to the administration of etanercept ICV, the TNF inhibitory effects may have impacted additional cardio-regulatory regions in the brain and elicited a similar response as in the PVN, but in light of its central integrative function versus the other regions, the PVN was of utmost concern. Future studies should investigate these additional cardio-regulatory regions, as well as looking more specifically at the pathway between Ang II, TNF and the differential regulation of the RAS arms in the Ang II hypertension-induced animal model. Our findings provide further evidence and insight for the involvement of the RAS within the PVN and its interaction and mediation through TNF in the neurogenic component of hypertension. Further exploration of these system interactions within the brain may be beneficial towards the development of novel hypertensive therapeutics.
